# Identification of tumor mutation burden-associated molecular and clinical features in cancer by analyzing multi-omics data

**DOI:** 10.3389/fimmu.2023.1090838

**Published:** 2023-02-24

**Authors:** Mengyuan Li, Xuejiao Gao, Xiaosheng Wang

**Affiliations:** ^1^ School of Pharmacy, Nanjing University of Chinese Medicine, Nanjing, China; ^2^ Affiliated Hospital of Integrated Traditional Chinese and Western Medicine, Nanjing University of Chinese Medicine, Nanjing, Jiangsu, China; ^3^ School of Basic Medicine and Clinical Pharmacy, China Pharmaceutical University, Nanjing, China

**Keywords:** tumor mutation burden, multi-omics, antitumor immunity, cancer immunotherapy, TMB prognostic score, ceRNA

## Abstract

**Background:**

Tumor mutation burden (TMB) has been recognized as a predictive biomarker for immunotherapy response in cancer. Systematic identification of molecular features correlated with TMB is significant, although such investigation remains insufficient.

**Methods:**

We analyzed associations of somatic mutations, pathways, protein expression, microRNAs (miRNAs), long non-coding RNAs (lncRNAs), competing endogenous RNA (ceRNA) antitumor immune signatures, and clinical features with TMB in various cancers using multi-omics datasets from The Cancer Genome Atlas (TCGA) program and datasets for cancer cohorts receiving the immune checkpoint blockade therapy.

**Results:**

Among the 32 TCGA cancer types, melanoma harbored the highest percentage of high-TMB (≥ 10/Mb) cancers (49.4%), followed by lung adenocarcinoma (36.9%) and lung squamous cell carcinoma (28.1%). Three hundred seventy-six genes had significant correlations of their mutations with increased TMB in various cancers, including 11 genes (*ARID1A*, *ARID1B*, *BRIP1*, *NOTCH2*, *NOTCH4*, *EPHA5*, *ROS1*, *FAT1*, *SPEN*, *NSD1*,and *PTPRT*) with the characteristic of their mutations associated with a favorable response to immunotherapy. Based on the mutation profiles in three genes (*ROS1*, *SPEN*, and *PTPRT*), we defined the TMB prognostic score that could predict cancer survival prognosis in the immunotherapy setting but not in the non-immunotherapy setting. It suggests that the TMB prognostic score’s ability to predict cancer prognosis is associated with the positive correlation between immunotherapy response and TMB. Nine cancer-associated pathways correlated positively with TMB in various cancers, including nucleotide excision repair, DNA replication, homologous recombination, base excision repair, mismatch repair, cell cycle, spliceosome, proteasome, and RNA degradation. In contrast, seven pathways correlated inversely with TMB in multiple cancers, including Wnt, Hedgehog, PI3K-AKT, MAPK, neurotrophin, axon guidance, and pathways in cancer. High-TMB cancers displayed higher levels of antitumor immune signatures and *PD-L1* expression than low-TMB cancers in diverse cancers. The association between TMB and survival prognosis was positive in bladder, gastric, and endometrial cancers and negative in liver and head and neck cancers. TMB also showed significant associations with age, gender, height, weight, smoking, and race in certain cohorts.

**Conclusions:**

The molecular and clinical features significantly associated with TMB could be valuable predictors for TMB and immunotherapy response and therefore have potential clinical values for cancer management.

## Introduction

Recently, cancer immunotherapies, including the immune checkpoint blockade (ICB) (1) and the chimeric antigen receptor (CAR) T cell therapy (2), have achieved notable success in treating various advanced malignancies. Nevertheless, only a subset of cancer patients responds to such treatments (3). Previous studies (4) have identified specific biomarkers for cancer immunotherapeutic responsiveness, including tumor PD-L1 expression levels (5), DNA mismatch-repair deficiency (6), tumor mutation burden (TMB) (7), and tumor-infiltrating lymphocytes (TILs) levels (8). In particular, the association between TMB and cancer immunotherapy response has been extensively investigated (7, 9–12). In general, high-TMB tumors tend to be more responsive to immunotherapy than low-TMB tumors and therefore have a better prognosis in the immunotherapy setting (13). In contrast, high-TMB tumors often have a worse prognosis than low-TMB tumors in the non-immunotherapy setting (14). It suggests that the more active immunotherapy response in high-TMB tumors leads to a better prognosis versus low-TMB tumors in the immunotherapy setting.

Previous studies have associated certain molecular features with TMB in cancer (15). In a study of the TMB landscape across 100,000 human cancer genomes (15), Chalmers et al. identified 48 genes whose mutations were primarily associated with increased TMB in human cancers, among which there were many DNA mismatch repair pathway genes and DNA polymerase genes, including *MSH2*, *MSH6*, *MLH1*, *PMS2*, and *POLE*. In another exploration of the association between somatic genetic alterations and TMB in 513 non-small-cell lung cancers (NSCLC) (16), Zhu et al. found that *TP53* and *KRAS* mutations were significantly associated with high TMB, while *EGFR* mutations, *ALK* fusion, and *ROS1* fusion were significantly related to low TMB. Likewise, they found a strong association between alterations in DNA damage repair pathway genes and high TMB in NSCLC (16).

A common finding from these prior studies is that the microsatellite instability/deficient mismatch repair (MSI/dMMR) genomic feature correlates strongly with high TMB in cancer. However, Chalmers et al. revealed that only 16% of high-TMB tumors were characterized by the MSI/dMMR genomic feature (15). It suggests that there must be other molecular features significantly correlated with TMB. To identify TMB-associated molecular features, we analyzed multi-omics datasets for 32 cancer types from The Cancer Genome Atlas (TCGA) program (https://www.cancer.gov/about-nci/organization/ccg/research/structural-genomics/tcga). Based on the recently defined standard for high (≥ 10/Mb) and low (< 10/Mb) TMB for clinical application, we analyzed associations of TMB with gene mutations, protein expression, and pathway activity in 14 cancer types having more than ten high-TMB tumors. We reexamined associations of TMB with tumor immunity, immunotherapy response, prognosis, and various tumor phenotypes and clinical features in these cancer types. This study aimed to provide potential implications for what cause high TMB and what are the consequences of high TMB.

## Results

### Comparison of TMB between different cancer types

**Figure 1:**
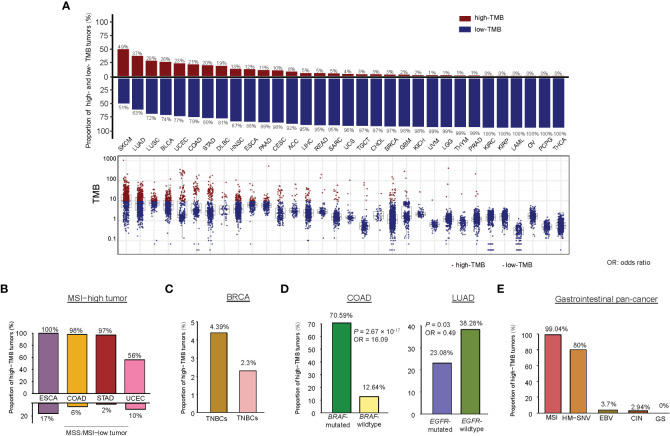
Comparison of TMB between different cancer types or subtypes. **(A)** The TMB distribution in each cancer type. Percentages of high-TMB cancers in each cancer type are shown. Proportions of high-TMB tumors in MSI-high versus MSS/MSI-low cancers **(B)**, in TNBC versus non-TNBC **(C)**, in COAD (or LUAD) with *BRAF* (or *EGFR*) mutations versus without *BRAF* (or *EGFR*) mutations **(D)**, and in five subtypes of gastrointestinal pan-cancer **(E)**.

Among the 32 cancer types, SKCM had the highest percentage of high-TMB cancers (49.4%), consistent with previous studies (14, 15). Two lung cancers had the second and third highest percentage of high-TMB cancers (36.9% and 28.1% for LUAD and LUSC, respectively). The other cancer types encompassing more than 10% of high-TMB cancers included BLCA, UCEC, COAD, STAD, DLBC, HNSC, ESCA, PAAD, and CESC (**Figure 1A**). The cancer types having less than 1% of high-TMB cancers included THYM, PRAD, KIRC, KIRP, LAML, OV, PCPG, and THCA. Since the high TMB is a biomarker for cancer immunotherapy response, the cancer types with a high percentage of high-TMB cancers would be suitable for immunotherapy. Indeed, immunotherapies, including ICB and CAR T cell therapy, have successfully treated skin, lung, bladder, head and neck, kidney, lymphoma, and MSI/dMMR cancers [10], most of which embraced more than 10% of high-TMB cancers except kidney cancer. The percentage of high-TMB cancers in pan-cancer was 11.5%, suggesting that around 11.5% of cancer patients are appropriate to immunotherapy in terms of the TMB criterion.

We examined TMB in different subtypes of a single cancer type. As expected, MSI-high tumors often had high TMB. For example, within the MSI-high subtype, 98% of COAD, 100% of ESCA, 97% of STAD, and 56% of UCEC were high-TMB tumors (**Figure 1B**). In contrast, within the microsatellite instability (MSS)/MSI-low subtype, only 6% of COAD, 17% of ESCA, 2% of STAD, and 10% of UCEC had high TMB. In BRCA, triple-negative breast cancers (TNBCs) had significantly higher TMB than non-TNBCs (one-tailed Mann-Whitney U test, *P* = 4.36 × 10^-11^) and harbored a higher proportion of high-TMB tumors (4.39% versus 2.30%) (**Figure 1C**). In addition, basal-like and HER2-enriched breast cancers had significantly higher TMB than luminal A&B (ER+) breast cancers (*P* < 0.001). In COAD, *BRAF*-mutated tumors had significantly higher TMB than *BRAF*-wildtype tumors (*P* = 1.37 × 10^-11^) and harbored a higher proportion of high-TMB tumors (70.59% versus 12.64%; *P* = 2.67 × 10^-17^, OR = 16.09) (**Figure 1D**). In LUAD, *EGFR*-mutated tumors had significantly lower TMB than *EGFR*-wildtype tumors (*P* = 2.13 × 10^-5^) and harbored a higher proportion of high-TMB tumors (23.08% versus 38.28%; *P* = 0.03, OR = 0.49) (**Figure 1D**). However, *KRAS*-mutated and *KRAS*-wildtype LUADs showed no significant difference in TMB (high-TMB tumors: 35.98% in *KRAS*-mutated versus 36.15% in *KRAS*-wildtype). In addition, we analyzed a gastrointestinal (GI) pan-cancer (17), which included 79 esophageal, 383 gastric, 341 colon, and 118 rectal cancers belonging to five subtypes: Epstein-Barr virus (EBV), MSI, hypermutated-SNV (HM-SNV), chromosomal instability (CIN), and genome stable (GS). We found that TMB followed the pattern: MSI&HM-SNV > CIN > GS&EBV (one-tailed Mann-Whitney U test, *P* < 0.05). The proportions of high-TMB tumors in these subtypes were 99.04%, 80%, 3.70%, 2.94%, and 0% for MSI, HM-SNV, EBV, CIN, and GS, respectively (**Figure 1E**).

### Identification of genes whose mutations correlate with TMB in cancer

**Figure 2:**
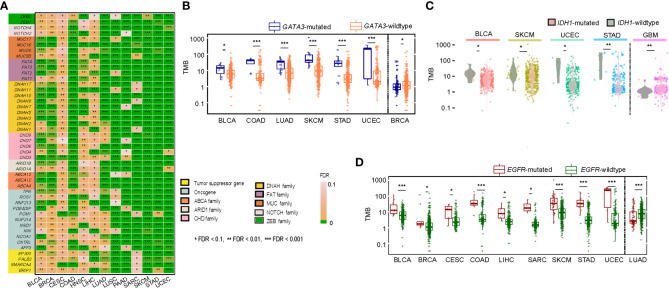
Correlations of gene mutations with TMB. **(A)** A list of tumor suppressor genes, oncogenes, and gene families whose mutations were significantly associated with increased TMB in at least ten cancer types. Comparisons of TMB between *GATA3*-mutated and *GATA3*-wildtype **(B)**, between *IDH1*-mutated and *IDH*-wildtype **(C)**, and between *EGFR*-mutated and *EGFR*-wildtype cancers **(D)**. The one-tailed Mann-Whitney U test adjusted *P*-values (FDR) are indicated.

We identified 376 genes whose mutations significantly correlated with increased TMB in at least 10 of the 14 cancer types (one-tailed Mann-Whitney U test, false discovery rate (FDR) < 0.1) (**Supplementary Table S1A**). These genes included some critical tumor suppressor genes (such as *BRIP1*, *SMARCA4*, *PALB2*, and *EP300*) and oncogenes (such as *AFF3*, *CNTRL*, *NCOA1*, *NIN*, *NOTCH2*, *NSD1*, *NUP214*, *PCM1*, *PDE4DIP*, *RNF213*, *ROS1*, and *TPR*) (**Figure 2A**). Notably, many of these genes belong to the same families, including *ABCA* (*ABCA4*, *12*&*13*), *ARID1* (*ARID1A*&*B)*, *CHD* (*CHD3*, *4*, *6*, *7*&*9*), *DNAH (DNAH1*, *2*, *3*, *5*, *7*, *8*, *10*, *11*&*17)*, *FAT* (*FAT1*, *2*, *3*&*4)*, *MUC* (*MUC5B*, *6*, *16*&*17*), *NOTCH* (*NOTCH2*&*4*), and *ZEB* (*ZEB1*&*2*). GSEA (18) revealed that these genes were significantly associated with the pathways regulating cellular activity, including focal adhesion, ECM-receptor interaction, calcium signaling, tight junction, gap junction, and ABC transporters. Also, we found 28 genes whose mutations correlated with reduced TMB in a single cancer type (**Supplementary Table S1B**). For example, *GATA3* mutations connected with reduced TMB in BRCA and increased TMB in five other cancer types (**Figure 2B**). *IDH1* mutations were associated with reduced TMB in GBM. They increased TMB in four different cancer types (**Figure 2C**). *EGFR* mutations were associated with reduced TMB in LUAD and increased TMB in nine other cancer types (**Figure 2D**). The negative correlation between mutations of these genes and TMB may explain why their mutations are associated with a better prognosis in these cancer types (19–21). Overall, these results indicate that the association between mutations in a single gene and TMB is likely positive in cancer. However, a negative association between them may occur in a few cases.

**Figure 3:**
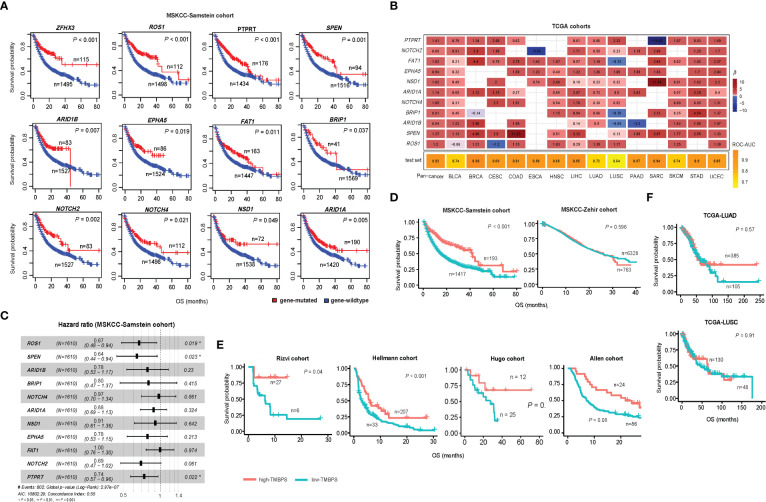
Correlations of gene mutations with survival prognosis in cancer. **(A)** Kaplan-Meier curves showing 12 genes whose mutations correlate with better overall survival (OS) in the MSKCC-Samstein cohort receiving the immune checkpoint blockade (ICB) therapy. The 12 genes also significantly correlate their mutations with increased TMB in at least 10 TCGA cancer types. **(B)** Predictions of high- versus low-TMB in TCGA pan-cancer and in 14 individual cancer types based on the mutation status of 11 genes using logistic regression models with the variables selected by the Lasso. The β-values for each variable in the logistic regression models and the ROC-AUC indicate the prediction performance. **(C)** Correlations between mutations of 11 genes and OS in the MSKCC-Samstein cohort by the Cox proportional hazards model. **(D)** Kaplan-Meier curves showing that the TMB prognostic score (TMBPS) correlates positively with OS in the MSKCC-Samstein cohort receiving the ICB therapy, while it shows no significant correlation with OS in the MSKCC-Zehir cohort without the ICB therapy. **(E)** Kaplan-Meier curves show that the TMBPS correlates positively with OS in two lung cancer cohorts (Rizvi and Hellmann cohorts) and two melanoma cohorts (Allen and Hugo cohort) receiving the ICB therapy. **(F)** In contrast, it shows no significant correlation with OS in the TCGA lung cancers not receiving the ICB therapy. The log-rank test *P*-values are shown in the survival analyses.

Since high TMB is associated with a favorable immunotherapy response in cancer patients (13), we anticipated that the patients harboring mutations in any of the 376 genes would have better survival than those without such mutations. As expected, among the 21 genes overlapping between the 376 genes and the 474 genes studied in the pan-cancer cohort [MSKCC-Samstein cohort (13)] receiving the ICB therapy, 12 genes exhibited a significant correlation of their mutations with better overall survival (OS) in the MSKCC-Samstein cohort (log-rank test, *P* < 0.05) (**Figure 3A**). The 12 genes included *ARID1A*, *ARID1B*, *BRIP1*, *NOTCH2*, *NOTCH4*, *EPHA5*, *ROS1*, *FAT1*, *SPEN*, *NSD1*, *PTPRT*, and *ZFHX3*. Among them, only the mutation of *ZFHX3* was associated with better OS in another pan-cancer cohort [MSKCC-Zehir cohort (13)] not receiving the ICB therapy (*P* = 0.007) (**Supplementary Figure 1**). It indicated that 11 of the 12 genes (except *ZFHX3*) had the characteristic of their mutations correlated with both high TMB and a favorable response to immunotherapy in cancer. Next, we used logistic models to explore the ability of mutations in the 11 genes to predict high- versus low-TMB in TCGA pan-cancer and the 14 individual cancer types. We first utilized the least absolute shrinkage and selection operator (Lasso) (22) to select variables (genes) and refitted logistic regression models with the variables selected by the Lasso. The Lasso selected all 11 genes in the pan-cancer analysis, and in analyzing individual cancer types, each gene was selected by the Lasso at least eight times (**Figure 3B**). The prediction accuracy was 92% in both training and test sets in the pan-cancer analysis and more than 80% in 10 individual cancer types. Furthermore, we analyzed the correlations between mutations of the 11 genes and OS in the MSKCC-Samstein cohort using the Cox proportional hazards model. We found three genes (*ROS1*, *SPEN*, and *PTPRT*) whose mutations had a significant association with better OS (*ROS1*: *P* = 0.019, HR = 0.67, 95% CI: [0.48, 0.94]; *SPEN*: *P* = 0.023, HR = 0.64, 95% CI: [0.44, 0.94]; *PTPRT*: *P* = 0.022, HR = 0.74, 95% CI: [0.57, 0.96]) (**Figure 3C**). We reanalyzed the correlations between mutations of the three genes and OS in the MSKCC-Samstein cohort using the Cox proportional hazards model and obtained their coefficients. Based on the mutation status of the three genes, we defined the TMB prognostic score (TMBPS) as: TMBPS = - (*a* × mutated (*ROS1*) + *b* × mutated (*SPEN*) + *c* × mutated (*PTPRT*)), where *a =* -0.5071, *b = -*0.5189, and *c =* -0.3485, which were the coefficients of the three variables in the Cox proportional hazards model*;* mutated(*X*) = 1 if the gene *X* is mutated in the tumor sample, otherwise mutated(*X*) = 0. We found that high-TMBPS (> median) tumors showed better OS than low-TMBPS tumors in the MSKCC-Samstein cohort (*p* = 1.85 × 10^-9^) (**Figure 3D**), but high- and low-TMBPS tumors showed no significant different OS in the MSKCC-Zehir cohort without the ICB therapy (**Figure 3D**). We further confirmed that the TMBPS correlated positively with OS in two lung cancer cohorts [Rizvi cohort (10) and Hellmann cohort (23)] and two melanoma cohort [Hugo cohort (11) and Allen cohort (12)] receiving the ICB therapy (**Figure 3E**). In contrast, it showed no significant correlation with OS in the TCGA lung cancers (**Figure 3F**). These results demonstrate that the TMBPS can predict the response to ICB therapy.

### Identification of cancer-associated pathways significantly correlated with TMB in cancer

**Figure 4:**
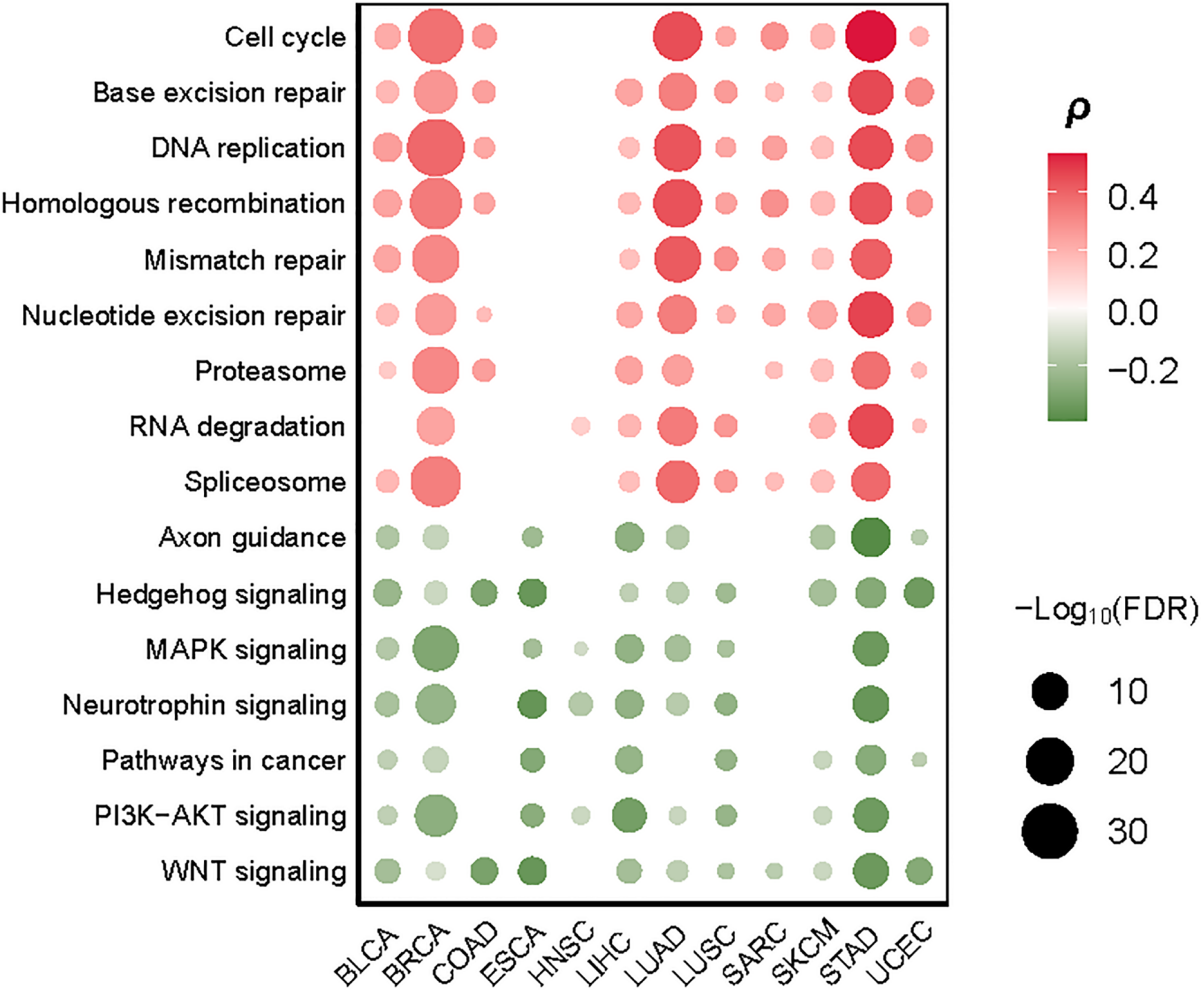
Cancer-associated pathways significantly correlate their activities with TMB in at least eight cancer types. The Spearman’s correlation test FDR and correlation coefficients (*ρ*) are indicated.

We identified nine cancer-associated pathways whose activity exhibited a significant positive correlation with TMB in at least eight cancer types (Spearman’s correlation test, FDR < 0.1). These pathways included nucleotide excision repair, DNA replication, homologous recombination, base excision repair, mismatch repair, cell cycle, spliceosome, proteasome, and RNA degradation (**Figure 4**). Notably, numerous DNA damage repair-associated pathways showed significant positive correlations with TMB (**Figure 4**). This is consistent with previous studies showing that dMMR has a strong correlation with high TMB in cancer (15). Interestingly, the cell cycle pathway showed a marked positive correlation with TMB in nine cancer types, including (BLCA, BRCA, COAD, LUAD, LUSC, SARC, SKCM, STAD, UCEC) (**Figure 4**), indicating that the proliferation of tumor cells increases TMB. The spliceosome pathway exhibited a significant correlation with TMB in eight cancer types (**Figure 4**); a possible explanation is that the spliceosome is associated with DNA damage repair and cell cycle progression (24). Moreover, we found seven pathways showing significant inverse correlations with TMB in at least eight cancer types (Spearman’s correlation test, FDR<0.1) (**Figure 4**). These pathways included Wnt, Hedgehog, PI3K-AKT, MAPK, neurotrophin, axon guidance, and pathways in cancer. Most of these pathways are overexpressed in cancer (25–29). Our data indicated that the hyperactivation of these pathways was associated with reduced TMB in cancer, suggesting that their activities inhibit the antitumor immune response. Interestingly, two neural development-associated pathways (neurotrophin and axon guidance) correlated significantly with TMB in diverse cancers. It suggests that the molecules involved in regulating neural development may play essential roles in modulating tumor immunity, as evidenced in a recent study (30).

### Identification of proteins whose expression correlates with TMB in cancer

**Figure 5:**
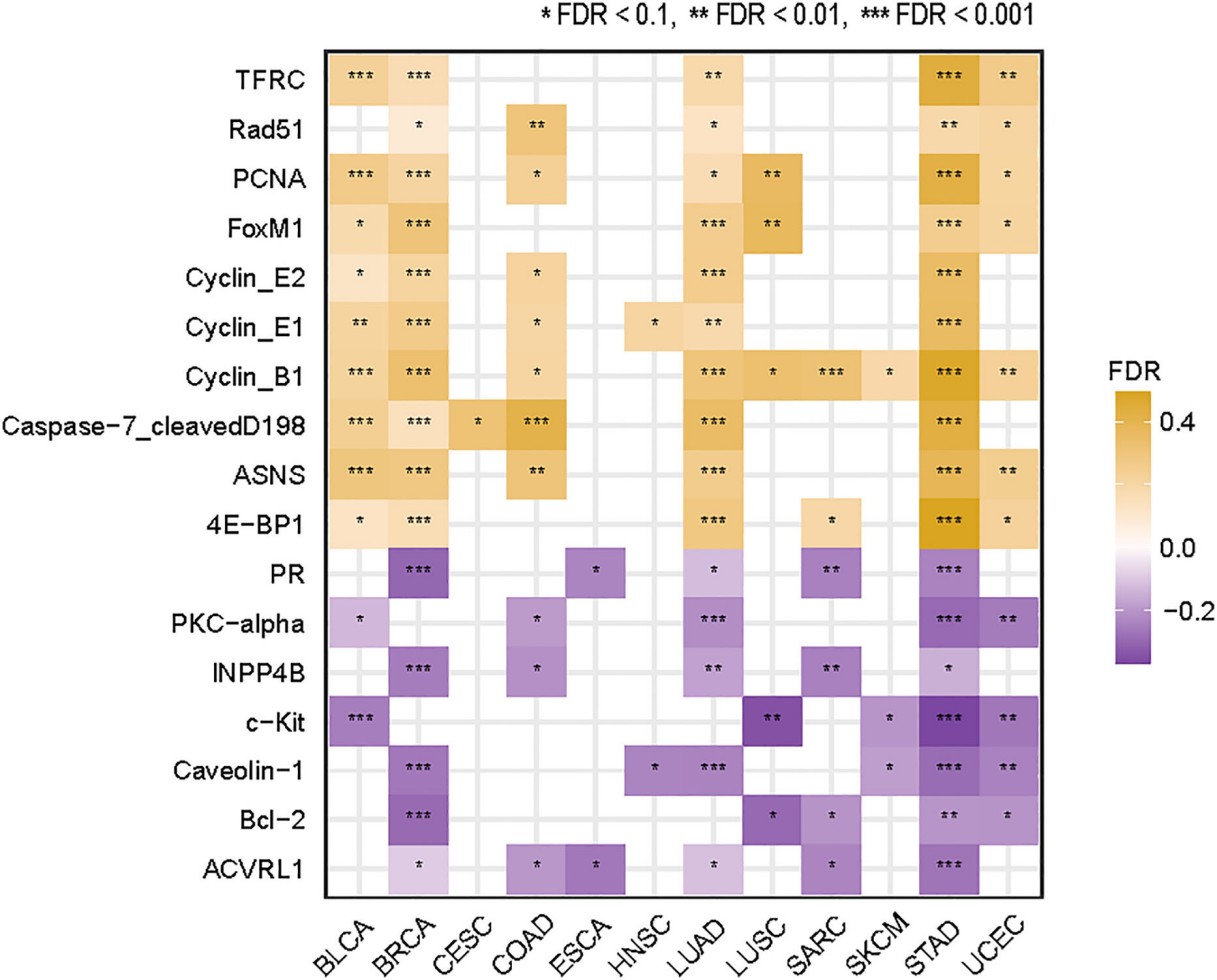
Proteins whose expression levels have significant correlations with TMB in at least five cancer types. The Spearman’s correlation test FDR and correlation coefficients (*ρ*) are indicated.

We identified ten proteins having significant positive expression correlations with TMB in at least five cancer types (Spearman’s correlation test, FDR < 0.1) (**Figure 5**). These proteins included ASNS, Cyclin_B1, Cyclin_E1, Cyclin_E2, Caspase-7, FoxM1, PCNA, Rad51, TFRC, and 4E-BP1. Many of these proteins were associated with cell cycle regulation, including ASNS, Cyclin_B1, Cyclin_E1, Cyclin_E2, and FoxM1. This is consistent with the fact that the cell cycle pathway correlates positively with TMB in multiple cancer types (**Figure 4**). Besides, several proteins were involved in the regulation of DNA damage repair, including PCNA and RAD51, consistent with the significant correlation between dMMR and high TMB in cancer. Furthermore, we identified seven proteins with significant inverse expression correlations with TMB in at least five cancer types (Spearman’s correlation test, FDR < 0.1) (**Figure 5**). These proteins included ACVRL1, Bcl-2, Caveolin-1, c-Kit, PKC-alpha, PR, and INPP4B. Among them, ACVRL1 is associated with the TGF-β pathway, which hampers antitumor immune response (31); Bcl-2 regulates the apoptosis pathway, which correlated inversely with TMB in four cancer types (**Figure 4**).

### Identification of ceRNA network which constructed by mRNA, miRNAs and lncRNAs whose expression correlates with TMB in cancer

**Figure 6:**
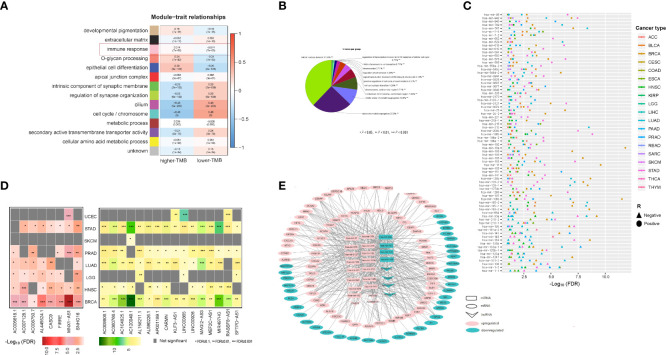
CeRNA network which constructed by mRNA, miRNAs and lncRNAs whose expression correlates with TMB in cancer. **(A)** WGCNA identified 14 coexpressed gene modules with softpower 7. The GO terms in the tan, black, pink, salmon, blue, red, green, greenyellow, magenta, turquoise, brown, purple, and yellow modules were mainly associated with developmental pigmentation, extracellular matrix, immune response, O-glycan processing, epithelial cell differentiation, apical junction complex, intrinsic component of synaptic membrane, regulation of synapse organization, cilium, cell cycle/chromosome, metabolic process, secondary active transmembrane transporter activity, and cellular amino acid metabolic process, respectively. **(B)** ClueGO mainly enriched the 107 WGCNA-DEmRNA in the mitotic nuclear division, regulation of cell division, and positive regulation of cell cycle phase transition. **(C)** 58 miRNAs whose expression levels were significantly higher in high-TMB cancers than in low-TMB cancers, and 18 miRNAs whose expression levels were significantly lower in high-TMB cancers than in low-TMB cancers in at least five cancer types (Student’s *t* test, FDR<0.1). **(D)** Left, eight lncRNAs whose expression levels were significantly higher in high-TMB cancers than in low-TMB cancers; Right, 16 lncRNAs whose expression levels were significantly lower in high-TMB cancers than in low-TMB cancers in at least five cancer types (Student’s *t* test, FDR<0.1). **(E)** CeRNA network constructed by 7 DElncRNAs, 20 DEmiRNAs, and 107 WGCNA-DEmRNA.

We identified 356 genes from the immune response module (pink module) which enriched in higher-TMB cancers and 674 genes from the cell cycle/chromosome module (turquoise module) which enriched in lower-TMB cancers among pan-cancer (WGCNA-pink-turquoise genes) by WGCNA method (**Figure 6A**). Meanwhile, we identified 730 and 248 mRNAs upregulated and downregulated differentially expressed mRNA genes (DEmRNA) between high-TMB cancers and low-TMB cancers in at least five cancer types, respectively (Student’s *t* test, FDR < 0.05, |FC| > 1.5). WGCNA-DEmRNA were defined as the intersection of WGCNA-pink-turquoise genes and DEmRNA. ClueGO mainly enriched the 107 WGCNA-DEmRNA in the mitotic nuclear division, regulation of cell division, and positive regulation of cell cycle phase transition (**Figure 6B**). Furthermore, we identified 58 (or 18) miRNAs and 8 (or 16) lncRNAs upregulated and downregulated differentially expressed miRNAs (DEmiRNA) and lncRNAs (DElncRNA) between high-TMB cancers and low-TMB cancers in at least five cancer types, respectively (Student’s *t* test, FDR<0.1) (**Figures 6C, D**). Many of these miRNAs were involved in the KEGG pathway MicroRNAs in cancer pathway (https://www.genome.jp/kegg-bin/show_pathway?hsa05206), including hsa-mir-192, hsa-mir-7-2, hsa-mir-96, hsa-mir-25, hsa-mir-7-3, hsa-mir-107, hsa-mir-128-2, hsa-mir-128-1, hsa-mir-183, hsa-mir-200b, hsa-mir-615, and hsa-mir-335. In addition, hsa-mir-25, hsa-mir-3074, and hsa-mir-106b were involved in the DNA damage response pathways. To further explore the molecular features of TMB, a competing endogenous RNAs (ceRNA) network was constructed and visualized by Cytoscape based on the intersection of 24 DElncRNA–DEmiRNA pairs and 383 WGCNA-DEmRNA–DEmiRNA pairs that were identified from the three databases [miRecords (32), miRTarBase (33, 34), starBase (35)]. (**Figure 6E**). The ceRNA network contains 7 DElncRNAs, 20 DEmiRNAs, and 107 WGCNA-DEmRNA.

### Association between TMB and antitumor immune response in cancer

**Figure 7:**
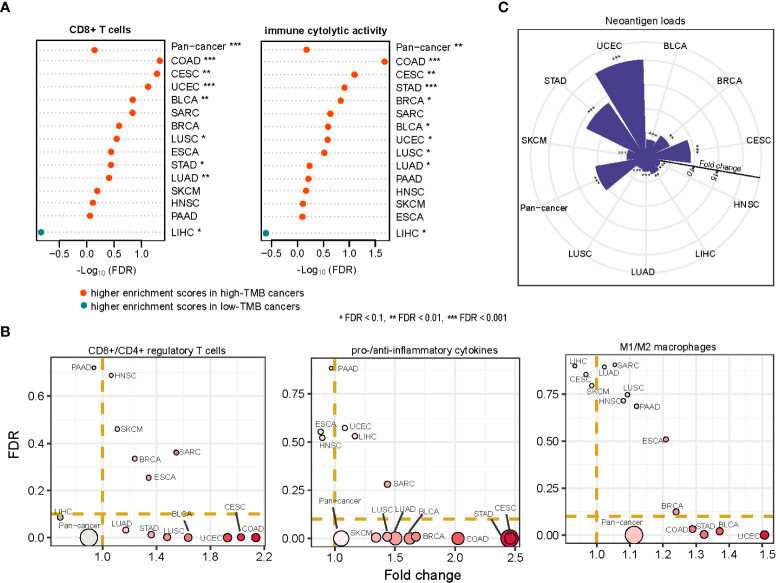
Association between TMB and antitumor immune response in cancer. The enrichment levels of antitumor immune signatures (CD8+ T cells and immune cytolytic activity) **(A)**, the ratios of immunostimulatory/immunoinhibitory signatures (CD8+/CD4+ regulatory T cells, pro-/anti-inflammatory cytokines, and M1/M2 macrophages) **(B)**, and the neoantigen loads **(C)** likely to be higher in high-TMB than in low-TMB cancers. The one-tailed Mann-Whitney U test **(A, C)** and two-tailed Student’s *t* test **(B)** FDRs are indicated.

It has been recognized that high-TMB cancers are likely to yield more neoantigens to incite antitumor immune responses (7). We found that the enrichment levels of CD8+ T cells were higher in high-TMB than in low-TMB cancers in seven cancer types (COAD, CESC, UCEC, BLCA, LUSC, STAD, LUAD). The immune cytolytic activity was higher in high-TMB cancers in eight cancer types (COAD, CESC, STAD, BRCA, BLCA, UCEC, LUSC, LUAD) (one-tailed Mann-Whitney U test, FDR < 0.1) (**Figure 7A**). Meanwhile, in 10 cancer types (COAD, STAD, LUAD, BLCA, SKCM, CESC, BRCA, UCEC, SARC, PAAD), high-TMB cancers displayed significantly higher *PD-L1* expression levels than low-TMB cancers (two-tailed Student’s *t* test, *P* < 0.1). It indicates that elevated TMB is associated with increases in both immunostimulatory and immunoinhibitory signatures. However, we found that the ratios of immunostimulatory/immunoinhibitory signatures (CD8+/CD4+ regulatory T cells, pro-/anti-inflammatory cytokines, and M1/M2 macrophages) were significantly higher in high-TMB than in low-TMB cancers (two-tailed Student’s *t* test, FDR < 0.1) in diverse cancer types (**Figure 7B**). The ratios were the average expression levels of immune-stimulatory signature marker genes over the average expression levels of immune-inhibitory signature marker genes. Furthermore, we found that high-TMB cancers encompassed more neoantigen loads than low-TMB cancers in all ten cancer types, in which the data of predicted neoantigen loads are available (**Figure 7C**) (36). These results confirmed that high TMB is associated with a more active antitumor immune response.

### Associations between TMB and clinical features in cancer

**Figure 8:**
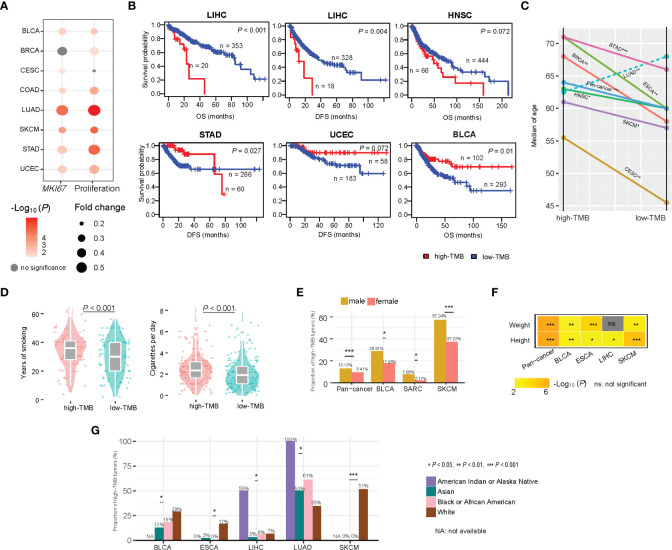
Associations between TMB and clinical features in cancer. **(A)** High-TMB cancers display significantly higher expression levels of *MKI67* and proliferation signature scores than low-TMB cancers in diverse cancers. **(B)** Kaplan-Meier curves show that the association between TMB and survival prognosis is cancer type dependent. Associations between TMB and age **(C)**, smoking **(D)**, gender **(E)**, weight and height **(F)**, and race **(G)** in cancer.

In seven of the 14 TCGA cancer types, high-TMB cancers displayed significantly higher expression levels of *MKI67*, a marker for tumor cell proliferation (37), and proliferation signature scores than low-TMB cancers (two-tailed Student’s *t* test, *P* < 0.1) (**Figure 8A**). It indicates that high TMB is associated with the increased proliferation potential of tumor cells. The association between TMB and survival prognosis is cancer type dependent. In LIHC and HNSC, high-TMB tumors had significantly worse survival (OS and/or disease-free survival (DFS)) prognosis than low-TMB tumors, while in BLCA, STAD, and UCEC, high-TMB tumors had significantly better survival prognosis (log-rank test, *P* < 0.1) (**Figure 8B** and **Supplementary Figure 2**).

We analyzed associations of TMB with age, gender, height, weight, smoking, and race. We found that the high-TMB patients were significantly older than the low-TMB patients in pan-cancer and in six individual cancer types, including BRCA, CESC, ESCA, HNSC, SKCM, and STAD (one-tailed Mann-Whitney U test, *P* < 0.1) (**Figure 8C**). This is reasonable because tumor mutations accumulate with age. One exception was LUAD, in which the high-TMB patients were significantly younger than the low-TMB patients (*P* < 0.001) (**Figure 8C**). The main reason could be that smoking significantly contributes to gene mutations during the development of lung cancer. Indeed, the smoking load had a significant positive correlation n with TMB in LUAD (*P* < 0.001) (**Figure 8D**). In pan-cancer and three individual cancer types, including BLCA, SARC, and SKCM, males had significantly higher proportions of high-TMB tumors than females (Fisher’s exact test, *P* < 0.1) (**Figure 8E**). However, in STAD, females had significantly higher proportions of high-TMB tumors than males (Fisher’s exact test, *P* < 0.002). In pan-cancer and four individual cancer types, including BLCA, ESCA, LIHC, and SKCM, the high-TMB patients were significantly higher than the low-TMB patients (one-tailed Mann-Whitney U test, *P* < 0.05) (**Figure 8F**). In addition, in pan-cancer and three individual cancer types, including BLCA, ESCA, and SKCM, the high-TMB patients were significantly heavier than the low-TMB patients (one-tailed Mann-Whitney U test, *P* < 0.01) (**Figure 8F**). Interestingly, in certain cancer types, TMB showed a significant association with race. In SKCM, ESCA, and BLCA, the proportion of high-TMB tumors in the white was significantly higher than that in other races (Chi-square test, *P* < 0.1) (**Figure 8G**). In contrast, in LUAD, the white harbored a significantly lower proportion of high-TMB tumors than other races (*P* = 0.01). In LIHC, the American Indian had the highest proportion (50%) of high-TMB tumors compared to other races (less than 10%).

## Discussion

TMB has been recognized as a biomarker for the response to immunotherapy in cancer (7, 13). Because whole-exome measures of TMB are often costly and have limited accuracy, TMB evaluated by targeted gene panel sequencing is clinically actionable (15). Certain genomic features, such as dMMR or MSI, have been indicated as valuable predictors of immunotherapy response for their strong correlations with TMB in cancer (6). In this study, we identified new molecular features correlated with TMB. First, we detected numerous genes whose mutations associated with increased TMB in diverse cancers, of which there were 11 genes whose mutations correlated with a favorable immunotherapy response. Of the 11 genes, some have been recognized for their significant association with immunotherapy response, such as *ARID1A* (38, 39). Previous studies demonstrated that the association between *ARID1A* mutations and favorable immunotherapy response was attributed to the increased TMB caused by *ARID1A* mutations (38, 39). This is consistent with our findings. Furthermore, based on the mutation profiles in three of the 11 genes (*ROS1*, *SPEN*, and *PTPRT*), we defined the TMB prognostic score that could predict cancer survival prognosis in the immunotherapy setting but not in the non-immunotherapy setting. It indicates that the TMB prognostic score’s ability to predict cancer prognosis is associated with the positive correlation between immunotherapy response and TMB. Then, we built an upgraded TMBPS (UTMBPS) score by molecular features (The mutation profiles in three genes (*ROS1*, *SPEN*, and *PTPRT*)) and clinical features. We incorporating the significant clinical covariants (gender) in multivariable Cox regression analysis in the MSKCC-Samstein cohort. We found three genes (*ROS1*, *SPEN*, and *PTPRT*) whose mutations and gender had a significant association with better OS (*ROS1*: *P* = 0.003, HR = 0.61, 95% CI: [0.44, 0.85]; *SPEN*: *P* = 0.007, HR = 0.60, 95% CI: [0.41, 0.87]; *PTPRT*: *P* = 0.0008, HR = 0.71, 95% CI: [0.55, 0.91], gender: *P* = 0.079, HR = 1.14, 95% CI: [0.99, 1.31]) (**Supplementary Figure 3A**). Based on the mutation status of the three genes and gender status, we defined the upgrade TMB prognostic score (UTMBPS) as: UTMBPS = - (*a* × mutated (*ROS1*) + *b* × mutated (*SPEN*) + *c* × mutated (*PTPRT*) + *d* × gender), where *a =* -0.7131, *b = -*0.7369, *c =* -0.4941, and *d* = 0.1890, which were the coefficients of the four variables in the Cox proportional hazards model: mutated(*X*) = 1 if the gene *X* is mutated in the tumor sample, otherwise mutated(*X*) = 0; gender(*X*) = 1 if the patient is female, otherwise gender(*X*) = 0. We found that high-UTMBPS (> median) tumors showed better OS than low-UTMBPS tumors in the MSKCC-Samstein cohort (*p* = 8.5 × 10^-9^) (**Supplementary Figure 3B**), but high- and low-UTMBPS tumors showed no significant different OS in the MSKCC-Zehir cohort without the ICB therapy (**Supplementary Figure 3B**).

Many DNA damage repair-associated pathways correlated with TMB, including nucleotide excision repair, DNA replication, homologous recombination, base excision repair, and mismatch repair. It is a fact that DNA damage repair deficiency may result in genomic instability, which in turn enhances TMB. Besides, the cell cycle activity correlated positively with TMB in multiple cancer types, suggesting that the growing tumor cell proliferation potential may raise TMB. This is in line with a recent study showing that increased cell cycle activity promoted an antitumor immune response in diverse cancers (40). The positive association between cell cycle activity and TMB suggests that combining cell cycle inhibitors with immunotherapy, as suggested in a recent study (41), could not be promising in improving the efficacy of cancer treatments. In addition, we found numerous oncogenic pathways whose activity correlated inversely with TMB, including the Wnt, Hedgehog, PI3K-AKT, and MAPK signaling pathways. This suggests that combining these pathways’ inhibitors and immunotherapy could improve the antitumor efficacy.

Our data showed that almost all the cancer types applying to immunotherapy harbored more than 10% of high-TMB tumors, including skin, lung, bladder, head and neck, lymphoma, and MSI/dMMR cancers. Among all cancer types, SKCM harbored the highest percentage of high-TMB tumors (49.4%), indicating that SKCM is the most suitable for immunotherapy. Indeed, current immune checkpoint inhibitors (ICIs) have achieved the earliest and most significant success in treating melanoma among all cancer types (42, 43). Next to SKCM were LUAD and LUSC, which included 36.9% and 28.1% of high-TMB tumors, respectively. Current ICIs have also successfully treated lung cancers (44–46). The MSI/dMMR cancers, which have high TMB and are prevalent in UCEC, COAD, and STAD, have been proven to have a favorable response to ICIs (47, 48). These results confirm that TMB is a valuable biomarker for predicting immunotherapy response.

Three kidney cancer types, including KIRC, KIRP, and KICH, harbored extremely low percentages of high-TMB tumors. Nevertheless, ICIs have been clinically used in treating kidney cancers (49), indicating that certain kidney cancers can respond to immunotherapy. The reason could be that many kidney cancers have high levels of immune cell infiltration (50). Taken together, these data indicate that TMB or neoantigens are not the only determinants for antitumor immunity and immunotherapy response. Thus, the discovery of other factors aside from TMB associated with antitumor immunity and immunotherapy response represents an interesting research direction.

## Conclusions

The molecular features significantly associated with TMB could be useful predictors for TMB and cancer immunotherapy response and therefore have potential clinical values for cancer therapy.

## Materials and methods

### Datasets

We downloaded gene somatic mutations (level 3), RNA-Seq gene expression profiles (level 3, RSEM-normalized), protein expression profiles (level 3), and clinical datasets for 32 cancer types from the TCGA data portal (https://portal.gdc.cancer.gov/). The cancer-associated pathways and genes involved in these pathways were obtained from KEGG (51). The somatic mutation profiles and clinical datasets for two other pan-cancer cohorts (MSKCC-Samstein and MSKCC-Zehir cohorts) were attained from their associated publication (13). In addition, we obtained the somatic mutation profiles and clinical datasets for two lung cancer cohorts (Rizvi cohort (10) and Hellmann cohort (23)) and two melanoma cohorts (Hugo cohort (11) and Allen cohort (12)) receiving ICB therapy from their associated publications.(**Supplementary Table 2**)

### Evaluation of TMB

For each tumor sample, its TMB was defined as the total count of non-synonymous somatic mutations/30Mb in the sample. We defined the high- and low-TMB according to the FDA standard. That is, if a tumor sample’s TMB is no less than 10, the tumor sample has a high-TMB. Otherwise, it has a low-TMB. The sample size for high- and low-TMB cancers is presented in **Supplementary Table S3**.

### Single-sample gene-set enrichment analysis

For a pathway, immune signature, or biological process, we quantified its enrichment level in a tumor sample as the single-sample gene-set enrichment analysis (ssGSEA) (52) score of its marker genes. The marker gene sets representing different immune signatures were from several publications, including CD8+ T cells (36), CD4+ regulatory T cells (36), pro-/anti-inflammatory cytokines (36), and M1/M2 macrophages immune cytolytic activity (36), and proliferation signature score (53). These gene sets are listed in **Supplementary Table S4**.

### Logistic regression analysis

We explored the ability of gene mutations to predict high- versus low-TMB cancers in TCGA pan-cancer and in 14 individual cancer types. We separated all samples into a training set (70% of the samples) and a test set (30% of the samples). On the training set, we used the Lasso to select variables with 10-fold cross-validation (CV) and refitted a logistic regression model with the variables selected by the Lasso using a 10-fold CV. We then tested the model on the test set and calculated the area under the curve of the receiver-operating characteristic (ROC-AUC) as the prediction performance. We performed the Lasso and logistic regression analyses using the R package “glmnet” and “caret,” respectively, and calculated the standardized regression coefficients (β values) using the function “lm. beta” in the R package “QuantPsyc.”

### Establishing the ceRNA network according to the DElncRNA–DEmiRNA–WGCNA–DEmRNA

The correlation between miRNA or lncRNA expression levels and TMB was assessed by comparing miRNA or lncRNA expression levels between high-TMB and low-TMB cancers in each caner type using Student’s *t* test. We identified miRNAs and lncRNAs whose expression levels were significantly higher (or lower) in high-TMB cancers than in low-TMB cancers in at least five cancer types as DEmiRNAs and DElncRNAs. We predicted DElncRNA–DEmiRNA pairs by the intersection of three databases [miRecords (32), miRTarBase (33, 34), starBase (35)] and using the R package “multiMiR”. WGCNA–DEmRNA–DEmiRNA pairs were identified by using starBase (http://starbase.sysu.edu.cn/) database. We establish and visualize the DElncRNA–DEmiRNA–WGCNA–DEmRNA ceRNA network based on the intersection of DElncRNA–DEmiRNA pairs and WGCNA–DEmRNA–DEmiRNA pairs (established by DElncRNAs, DEmiRNAs, and WGCNA–DEmRNA) by Cytoscape *v3.7.2* WGCNA analysis was carried out using R package "WGCNA".

We using the first 70% mRNAs by variance comparison to build the gene modules that were differentially enriched between the higher TMB level and the lower TMB level tumors in pan-cancer. Then, we identified 14 co-expressed gene modules with softpower 7. WGCNA-pink-turquoise genes was defined as genes which enriched in immune response and cell cycle modules (pink and turquoise modules). The interaction of WGCNA-pink-turquoise genes and DEmRNA was defined as WGCNA – DEmRNA. Cytoscape plug-in (ClueGO) (54) provides functional annotation for WGCNA – DEmRNA genes.

### Statistical analysis

In comparisons of two classes of data, we used the Mann-Whitney U test (for non-normally distributed data) or the Student’s *t* test (for normally distributed data). We used Spearman’s correlation test (for non-normally distributed data) or Pearson’s correlation test (for normally-distributed data) to evaluate the correlation between two groups of data. We used Fisher’s exact or Chi-square test to assess the association between two categorical variables. We utilized the FDR, evaluated by the Benjamini-Hochberg method, to adjust for multiple tests. We performed all statistical analyses in the R programming environment (version 4.0.2).

### Survival analysis

In the univariate survival analysis, we utilized the Kaplan-Meier curves to exhibit the survival time differences and the log-rank test to evaluate the significance of survival time differences. We performed the survival analyses using the function “survfit” in the R package “survival.” In the multivariate survival analysis, we used the cox proportional hazards model. We performed the multivariate survival analyses using the function “coxph” in R package “survival.”

## Data availability statement

The original contributions presented in the study are included in the article/**Supplementary Material**. Further inquiries can be directed to the corresponding author.

## Author contributions

ML performed the research and data analyses, designed methods, conceived this study, and wrote the manuscript. XG performed the research and data analyses. XW designed methods, conceived this study and wrote the manuscript. All authors contributed to the article and approved the submitted version.

## Glossary

**Table:**

TMB	tumor mutation burden
TCGA	The Cancer Genome Atlas
TILs	tumor-infiltrating lymphocytes
NSCLC	non-small-cell lung cancers
MSI	microsatellite instability
dMMR	deficient mismatch repair
PD-L1	programmed cell death one ligand
FDR	false discovery rate
FAT	FAT atypical cadherin.
MUC	mucin
IDH1	isocitrate dehydrogenase 1
ssGSEA	the single-sample gene-set enrichment analysis
BLCA	bladder urothelial carcinoma
BRCA	breast invasive carcinoma
CHOL	cholangiocarcinoma
COAD	colon adenocarcinoma
ESCA	esophageal carcinoma
GBM	glioblastoma multiforme
HNSC	head and neck squamous cell carcinoma
KICH	kidney chromophobe
KIRC	kidney renal clear cell carcinoma
KIRP	kidney renal papillary cell carcinoma
LIHC	liver hepatocellular carcinoma
LUAD	lung adenocarcinoma
LUSC	lung squamous cell carcinoma
PRAD	prostate adenocarcinoma
READ	rectum adenocarcinoma
STAD	stomach adenocarcinoma
THCA	thyroid carcinoma
UCEC	uterine corpus endometrial carcinoma
ACC	adrenocortical carcinoma
CESC	cervical squamous-cell carcinoma and endocervical adeno-carcinoma
LAML	acute myeloid leukemia
LGG	brain lower-grade glioma
OV	ovarian serous cystadenocarcinoma
PAAD	pancreatic adeno-carcinoma
SARC	sarcoma
SKCM	skin cutaneous melanoma
TGCT	testicular germ-cell tumors
UCS	uterine carcino-sarcoma
UVM	uveal melanoma
THYM	thymoma
DLBC	lymphoid neoplasm diffuse large B-cell lymphoma
PCPG	pheochromocytoma and paraganglioma
TMBPS	TMB prognostic score.
miRNAs	microRNAs
lncRNAs	long non-coding RNAs.
