# Supplementary material for: Identification of tumor mutation burden-associated molecular and clinical features in cancer by analyzing multi-omics data
Source: Front Immunol. 2023 Feb 24;14:1090838. doi: 10.3389/fimmu.2023.1090838 (PMC9998480; doi:10.3389/fimmu.2023.1090838)
Supplement: Supplementary file 1 [file DataSheet_1.pdf]

Supplementary Materials

Supplementary Table 1. genes whose mutations were significantly correlated with TMB. A. 376 genes whose mutations were significantly correlated with increased TMB in at least 10 cancer types. **B.** 28 genes whose mutations correlated with reduced TMB in a single cancer type.

Supplementary Table 2. A summary of datasets used in this study.

Supplementary Table 3. The sample size for high-TMB and low-TMB cancers.

Supplementary Table 4. The marker genes of immune signatures and proliferation.

Supplementary Figure 1. Kaplan-Meier curves show that the mutations in certain genes (expect *ZFHX3*) whose mutations were significantly associated with increased TMB have no significant correlation with overall survival in pan-cancer (MSKCC-Zehir cohort) not receiving the ICB therapy.

Supplementary Figure 2. The association between TMB and survival prognosis in different cancer types.

Supplementary Figure 3. Correlations between mutations of three genes, gender and OS. A. Cox proportional hazards model in the MSKCC-Samstein cohort. **B**. Kaplan-Meier curves showing that the upgraded TMB prognostic score (UTMBPS) correlates positively with OS in the MSKCC-Samstein cohort receiving the ICB therapy, while it shows no significant correlation with OS in the MSKCC-Zehir cohort without the ICB therapy.

Table S1A. 376 genes whose mutations were significantly correlated with increased TMB in at least 10 cancer types.

Gene symbol	BLCA	BRCA	CESC	COAD	ESCA	HNSC	LIHC	LUAD	LUSC	PAAD	SARC	SKCM	STAD	UCEC	Total
TTN	1	1	1	1	1	1	1	1	1	0	1	1	1	1	13
LRP1B	1	1	1	1	1	1	1	1	1	0	1	1	1	1	13
GPR98	1	1	1	1	0	1	1	1	1	0	1	1	1	1	12
SYNE1	1	1	1	1	0	1	1	1	1	0	1	1	1	1	12
PKHD1L1	1	1	1	1	0	1	1	1	1	0	1	1	1	1	12
ITPR2	1	1	1	1	0	1	1	1	0	1	1	1	1	1	12
RYR1	1	1	1	1	0	1	1	1	0	1	1	1	1	1	12
NAV3	1	1	1	1	0	1	1	1	1	0	1	1	1	1	12
RYR3	1	1	1	1	0	1	1	1	1	0	1	1	1	1	12
OBSCN	1	1	1	1	0	1	1	1	1	0	1	1	1	1	12
FCGBP	1	1	1	1	1	1	1	1	0	0	1	1	1	1	12
USH2A	1	1	1	1	0	1	1	1	1	0	1	1	1	1	12
HMCN1	1	1	1	1	0	1	1	1	1	0	1	1	1	1	12
ABCA13	1	1	1	1	0	1	1	1	1	1	0	1	1	1	12
PCDH15	1	1	1	1	0	1	1	1	1	1	0	1	1	1	12
XIRP2	1	1	1	1	0	1	1	1	1	0	1	1	1	1	12
AHNAK2	1	1	1	1	0	1	1	1	1	0	1	1	1	1	12
PEG3	1	1	1	1	0	1	1	1	1	0	1	1	1	1	12
MGAM	1	1	1	1	0	1	1	1	1	0	1	1	1	1	12
ZFHX3	1	1	1	1	0	1	1	1	0	1	1	1	1	1	12
ZFHX4	1	1	1	1	0	1	1	1	1	0	1	1	1	1	12

SCN11A	1	1	1	1	0	1	1	1	1	0	1	1	1	1	12
ADAMTS3	1	1	1	1	0	1	1	1	1	0	1	1	1	1	12
SFMBT2	1	1	1	1	0	1	1	1	0	1	1	1	1	1	12
MUC16	1	1	1	1	0	1	1	1	0	0	1	1	1	1	11
DST	1	1	1	1	0	1	1	1	1	0	0	1	1	1	11
AHNAK	1	1	1	1	0	1	1	1	0	0	1	1	1	1	11
CHD7	1	1	1	1	0	1	1	1	0	1	0	1	1	1	11
SYNE2	1	1	1	1	0	1	1	1	1	0	0	1	1	1	11
DNAH8	1	1	1	1	0	1	1	1	0	0	1	1	1	1	11
FAT2	1	1	1	1	0	1	1	1	1	0	0	1	1	1	11
NEB	1	1	1	1	0	1	1	1	1	0	0	1	1	1	11
DNAH7	1	1	1	1	0	1	1	1	0	1	0	1	1	1	11
PDE4DIP	1	1	1	1	0	1	1	1	0	1	0	1	1	1	11
LAMA3	1	1	1	1	0	1	1	1	0	0	1	1	1	1	11
PCNT	1	1	1	1	0	1	1	1	0	0	1	1	1	1	11
FRAS1	1	1	1	1	0	1	1	1	0	0	1	1	1	1	11
UTRN	1	1	1	1	0	1	1	1	1	0	0	1	1	1	11
APOB	1	1	1	1	0	1	0	1	1	0	1	1	1	1	11
MUC17	1	1	1	1	0	1	1	1	1	0	0	1	1	1	11
DIDO1	1	1	1	1	0	1	1	1	1	0	0	1	1	1	11
ASXL2	1	1	1	1	0	1	1	1	0	1	0	1	1	1	11
CEP350	1	1	1	1	0	1	0	1	1	0	1	1	1	1	11
MDN1	1	1	1	1	0	1	1	1	1	0	0	1	1	1	11
CSMD3	1	1	1	1	0	1	1	1	1	0	0	1	1	1	11
PLEC	1	1	1	1	0	1	1	1	0	0	1	1	1	1	11

CKAP5	1	1	1	1	0	1	1	1	0	1	0	1	1	1	11
TPR	1	1	1	1	0	1	1	1	1	0	0	1	1	1	11
IGSF10	1	1	1	1	0	1	1	1	0	0	1	1	1	1	11
TMEM131	1	1	1	1	0	1	1	1	0	1	0	1	1	1	11
LRP2	1	1	1	1	0	1	1	1	1	0	0	1	1	1	11
DNAH17	1	1	1	1	0	1	1	1	0	0	1	1	1	1	11
GON4L	1	1	1	1	0	1	0	1	1	1	0	1	1	1	11
SI	1	1	1	1	0	1	1	1	1	0	0	1	1	1	11
EIF4G1	1	1	1	1	0	1	1	1	0	1	0	1	1	1	11
SIPA1L1	1	1	1	1	0	1	1	1	0	1	0	1	1	1	11
PCDH10	1	1	1	1	0	1	0	1	0	1	1	1	1	1	11
FBN2	1	1	1	1	0	1	1	1	1	0	0	1	1	1	11
ANK2	1	1	1	1	0	1	1	1	0	0	1	1	1	1	11
MYO7A	1	1	1	1	0	1	1	1	0	0	1	1	1	1	11
UNC79	1	1	1	1	0	1	1	1	1	0	0	1	1	1	11
EPPK1	1	1	1	1	0	1	1	1	0	0	1	1	1	1	11
PDZD2	1	1	1	1	0	1	1	1	0	1	0	1	1	1	11
USP34	1	1	1	1	0	1	1	1	1	0	0	1	1	1	11
PLXNA4	1	1	1	1	0	1	1	1	1	0	0	1	1	1	11
TECTA	1	1	1	1	0	1	1	1	0	0	1	1	1	1	11
DMBT1	1	1	1	1	0	1	0	1	0	1	1	1	1	1	11
ASXL3	1	1	1	1	0	1	1	1	0	0	1	1	1	1	11
FLG2	1	1	1	1	0	1	1	1	0	0	1	1	1	1	11
RYR2	1	1	1	1	0	1	1	1	1	0	0	1	1	1	11
CUBN	1	1	1	1	0	1	1	1	0	0	1	1	1	1	11

LAMA2	1	1	1	1	0	1	1	1	0	0	1	1	1	1	11
MYO7B	1	1	1	1	0	1	1	1	0	0	1	1	1	1	11
DCHS1	1	1	1	1	0	1	1	1	1	0	0	1	1	1	11
MAP2	1	1	1	1	0	1	1	1	0	0	1	1	1	1	11
PCLO	1	1	1	1	0	1	1	1	0	0	1	1	1	1	11
RP1	1	1	1	1	0	1	1	1	0	0	1	1	1	1	11
CACNA1B	1	1	1	1	0	1	1	1	0	0	1	1	1	1	11
ROS1	1	1	1	1	0	1	1	1	1	0	0	1	1	1	11
CROCC	1	1	1	1	0	1	1	1	0	1	0	1	1	1	11
SUPT6H	1	1	1	1	0	1	1	1	0	1	0	1	1	1	11
DOCK10	1	1	1	1	0	1	1	1	1	0	0	1	1	1	11
FBN3	1	1	1	1	0	1	1	1	0	0	1	1	1	1	11
CDH10	1	1	0	1	0	1	1	1	0	1	1	1	1	1	11
VCAN	1	1	1	1	0	1	1	1	0	0	1	1	1	1	11
CCDC108	1	1	1	1	0	1	0	1	1	0	1	1	1	1	11
PCNX	1	1	1	1	0	1	1	1	1	0	0	1	1	1	11
SPHKAP	1	1	1	1	0	1	0	1	1	0	1	1	1	1	11
MACF1	1	1	1	1	0	1	1	1	0	0	0	1	1	1	10
FAT4	1	1	1	1	0	1	1	1	0	0	0	1	1	1	10
DNAH3	1	1	1	1	0	1	1	1	0	0	0	1	1	1	10
LAMA1	1	1	1	1	0	1	1	1	0	0	0	1	1	1	10
CEP250	1	1	0	1	0	1	1	1	0	0	1	1	1	1	10
DMXL2	1	1	1	1	0	0	1	1	1	0	0	1	1	1	10
VPS13D	1	1	1	1	0	1	1	1	0	0	0	1	1	1	10
DOCK9	1	1	1	1	0	1	1	1	0	0	0	1	1	1	10

PKHD1	1	1	0	1	0	1	1	1	1	0	0	1	1	1	10
POLQ	1	1	1	1	0	1	1	1	0	0	0	1	1	1	10
SPEN	1	1	1	1	0	1	1	1	0	0	0	1	1	1	10
ACAN	1	1	1	1	0	1	0	1	0	0	1	1	1	1	10
LAMB1	1	1	1	1	0	1	1	1	0	0	0	1	1	1	10
MYCBP2	1	1	1	1	0	1	1	1	0	0	0	1	1	1	10
C2orf16	1	1	1	1	0	0	1	1	1	0	0	1	1	1	10
NCOA1	1	1	1	1	0	1	1	1	0	0	0	1	1	1	10
DOPEY2	1	1	1	1	0	1	1	1	0	0	0	1	1	1	10
MYH15	1	1	1	1	0	1	0	1	0	0	1	1	1	1	10
KIAA1109	1	1	1	1	0	1	1	1	0	0	0	1	1	1	10
ABCA12	1	1	1	1	0	1	1	1	0	0	0	1	1	1	10
KALRN	1	1	1	1	0	1	1	1	0	0	0	1	1	1	10
DNAH10	1	1	1	1	0	1	1	1	0	0	0	1	1	1	10
MAP3K5	1	1	1	1	0	1	1	1	0	0	0	1	1	1	10
LRRIQ1	1	1	1	1	0	1	1	1	0	0	0	1	1	1	10
ALMS1	1	1	1	1	0	1	0	1	1	0	0	1	1	1	10
TACC2	1	1	1	1	0	1	1	1	0	0	0	1	1	1	10
VPS13C	1	1	1	1	0	1	1	1	0	0	0	1	1	1	10
BAZ2B	1	1	1	1	0	1	0	1	0	0	1	1	1	1	10
ANK3	1	1	1	1	0	1	1	1	0	0	0	1	1	1	10
SRCAP	1	1	1	1	0	1	1	1	0	0	0	1	1	1	10
SPAG17	1	1	1	1	0	1	1	1	0	0	0	1	1	1	10
KIAA0947	1	1	1	1	0	1	1	1	0	0	0	1	1	1	10
RNF213	1	1	1	1	0	1	0	1	1	0	0	1	1	1	10

SACS	1	1	1	1	0	1	1	1	0	0	0	1	1	1	10
DNAH11	1	1	1	1	0	1	0	1	1	0	1	1	0	1	10
ITSN2	1	1	1	1	0	1	0	1	1	0	0	1	1	1	10
DNAH5	1	1	1	1	0	1	1	1	0	0	0	1	1	1	10
TRANK1	1	1	0	1	0	1	1	1	0	0	1	1	1	1	10
FAT3	1	1	1	1	0	1	1	1	0	0	0	1	1	1	10
CDC42BPG	1	1	1	1	0	1	1	1	0	0	0	1	1	1	10
SPTBN5	1	1	1	1	0	1	1	1	0	0	0	1	1	1	10
HUWE1	1	1	1	1	0	1	1	1	0	0	0	1	1	1	10
CHD9	1	1	1	1	0	1	1	1	0	0	0	1	1	1	10
TNRC6B	1	1	1	1	0	1	1	1	0	0	0	1	1	1	10
FAM208B	1	1	1	1	0	1	1	1	0	0	0	1	1	1	10
CHD6	1	1	1	1	0	1	1	1	0	0	0	1	1	1	10
ACACA	1	1	1	1	0	1	1	1	0	0	0	1	1	1	10
KIAA2026	1	1	1	1	0	1	1	1	0	0	0	1	1	1	10
MYH10	1	1	1	1	0	1	1	1	0	0	0	1	1	1	10
ARID1B	1	1	1	1	0	1	1	1	0	0	0	1	1	1	10
FREM2	1	1	1	1	0	1	1	1	0	0	0	1	1	1	10
DLEC1	1	1	1	1	0	1	1	1	0	0	0	1	1	1	10
NLRC5	1	1	1	1	0	1	1	1	0	0	0	1	1	1	10
SYTL2	1	1	1	1	0	1	1	1	0	0	0	1	1	1	10
COL6A6	1	1	1	1	0	1	0	1	0	0	1	1	1	1	10
CEP192	1	1	1	1	0	1	1	1	0	0	0	1	1	1	10
KIDINS220	1	1	1	1	0	1	1	1	0	0	0	1	1	1	10
SCN2A	1	1	1	1	0	1	0	1	1	0	0	1	1	1	10

REV3L	1	1	0	1	0	1	1	1	1	0	0	1	1	1	10
SRRM2	1	1	1	1	0	1	1	1	0	0	0	1	1	1	10
SZT2	1	1	1	1	0	1	1	1	0	0	0	1	1	1	10
TRRAP	1	1	1	1	0	1	1	1	0	0	0	1	1	1	10
COL12A1	1	1	0	1	1	1	1	1	0	0	0	1	1	1	10
BSN	1	1	1	1	0	1	1	1	0	0	0	1	1	1	10
MUC6	1	1	1	1	0	1	1	1	0	0	0	1	1	1	10
MAP1A	1	1	1	1	0	1	1	1	0	0	0	1	1	1	10
TEP1	1	1	1	1	0	1	1	1	0	0	0	1	1	1	10
PTPN13	1	1	1	1	0	1	1	1	0	0	0	1	1	1	10
NCOR1	1	1	1	1	0	1	1	1	0	0	0	1	1	1	10
SLITRK3	1	1	0	1	0	1	1	1	1	0	0	1	1	1	10
FASN	1	1	1	1	0	1	1	1	0	0	0	1	1	1	10
PHLDB2	1	1	0	1	0	1	1	1	0	0	1	1	1	1	10
ABCA4	1	1	1	1	0	1	1	1	0	0	0	1	1	1	10
VPS13B	1	1	1	1	0	1	0	1	1	0	0	1	1	1	10
MYO18B	1	1	0	1	0	1	1	1	1	0	0	1	1	1	10
TNS3	1	1	1	1	0	1	1	1	0	0	0	1	1	1	10
TNIK	1	1	1	1	0	1	1	1	0	0	0	1	1	1	10
TCF20	1	1	1	1	0	1	0	1	0	1	0	1	1	1	10
KIAA0100	1	1	1	1	0	1	1	1	0	0	0	1	1	1	10
RIF1	1	1	1	1	0	1	1	1	0	0	0	1	1	1	10
HIVEP3	1	1	1	1	0	1	1	1	0	0	0	1	1	1	10
DSCAML1	1	1	0	1	0	1	1	1	1	0	0	1	1	1	10
EP400	1	1	1	1	0	1	0	1	0	0	1	1	1	1	10

USP9X	1	1	1	1	0	1	1	1	0	0	0	1	1	1	10
SIPA1L2	1	1	0	1	0	1	1	1	1	0	0	1	1	1	10
DYNC2H1	1	1	0	1	0	1	1	1	0	0	1	1	1	1	10
LRP1	1	1	0	1	0	1	1	1	0	1	0	1	1	1	10
CENPE	1	1	1	1	0	1	1	1	0	0	0	1	1	1	10
C6	1	1	1	1	0	1	0	1	1	0	0	1	1	1	10
DYNC1H1	1	1	1	1	0	0	1	1	1	0	0	1	1	1	10
RELN	1	1	0	1	0	1	1	1	0	0	1	1	1	1	10
TG	1	1	0	1	0	1	1	1	0	1	0	1	1	1	10
HFM1	1	1	1	1	0	1	1	1	0	0	0	1	1	1	10
LAMC1	1	1	0	1	0	1	1	1	0	0	1	1	1	1	10
SCN1A	1	1	0	1	0	1	1	1	1	0	0	1	1	1	10
EXOC4	1	1	1	1	0	1	1	1	0	0	0	1	1	1	10
C1orf173	1	1	0	1	0	1	1	1	1	0	0	1	1	1	10
SCN9A	1	1	1	1	0	1	0	1	0	0	1	1	1	1	10
RNF17	1	1	1	1	0	1	1	1	0	0	0	1	1	1	10
PLCL1	1	1	0	1	0	1	1	1	1	0	0	1	1	1	10
NIPBL	1	1	1	1	0	1	1	1	0	0	0	1	1	1	10
DMXL1	1	1	1	1	0	1	1	1	0	0	0	1	1	1	10
ARFGEF1	1	1	1	1	0	1	1	1	0	0	0	1	1	1	10
PI4KA	1	1	1	1	0	1	0	1	0	0	1	1	1	1	10
GOLGB1	1	1	1	1	0	1	0	1	1	0	0	1	1	1	10
EP300	1	1	1	1	0	1	0	1	0	0	1	1	1	1	10
GPR112	1	1	1	1	0	1	1	1	0	0	0	1	1	1	10
COL7A1	1	1	1	1	0	1	1	1	0	0	0	1	1	1	10

ZEB1	1	1	1	1	0	1	1	1	0	0	0	1	1	1	10
SNRNP200	1	1	1	1	0	0	1	1	0	0	1	1	1	1	10
LRRK2	1	1	0	1	1	1	1	1	0	0	0	1	1	1	10
CNTRL	1	1	1	1	0	1	1	1	0	0	0	1	1	1	10
C2CD3	1	1	1	1	0	1	1	1	0	0	0	1	1	1	10
IGSF22	1	1	1	1	0	1	1	1	0	0	0	1	1	1	10
WDHD1	1	1	0	1	0	1	1	1	1	0	0	1	1	1	10
CLSTN2	1	1	1	1	0	1	1	1	0	0	0	1	1	1	10
LRRK1	1	1	1	1	0	0	1	1	0	0	1	1	1	1	10
KIAA1429	1	1	1	1	0	1	1	1	0	0	0	1	1	1	10
DUSP27	1	1	1	1	0	1	1	1	0	0	0	1	1	1	10
CSPG4	1	1	1	1	0	1	1	1	0	0	0	1	1	1	10
CUX1	1	1	1	1	0	1	1	1	0	0	0	1	1	1	10
MED12L	1	1	1	1	0	1	1	1	0	0	0	1	1	1	10
ADCY2	1	1	1	1	0	1	1	1	0	0	0	1	1	1	10
NBEAL2	1	1	0	1	0	1	1	1	0	0	1	1	1	1	10
DPYD	1	1	1	1	0	1	0	1	0	0	1	1	1	1	10
ZZEF1	1	1	1	1	0	1	0	1	1	0	0	1	1	1	10
CHD4	1	1	1	1	0	1	0	1	0	1	0	1	1	1	10
ABCB1	1	1	1	1	0	1	0	1	1	0	0	1	1	1	10
COL11A1	1	1	0	1	0	1	1	1	1	0	0	1	1	1	10
CAMSAP2	1	1	1	1	0	1	1	1	0	0	0	1	1	1	10
DOCK2	1	1	1	1	0	1	1	1	0	0	0	1	1	1	10
DOCK8	1	1	1	1	0	1	1	1	0	0	0	1	1	1	10
HYDIN	1	1	1	1	0	1	1	1	0	0	0	1	1	1	10

ITGAM	1	1	1	1	0	1	0	1	1	0	0	1	1	1	10
OTOF	1	1	0	1	0	1	1	1	0	1	0	1	1	1	10
NUP153	1	1	1	1	0	1	1	1	0	0	0	1	1	1	10
FLNB	1	1	0	1	0	1	1	1	1	0	0	1	1	1	10
SEC24C	1	1	0	1	0	1	1	1	0	1	0	1	1	1	10
ANKRD26	1	1	1	1	0	1	1	1	0	0	0	1	1	1	10
ANKRD12	1	1	1	1	0	1	1	1	0	0	0	1	1	1	10
MUC5B	1	1	1	1	0	1	0	1	0	0	1	1	1	1	10
TNRC18	1	1	0	1	0	1	1	1	0	0	1	1	1	1	10
SPEF2	1	1	0	1	0	1	1	1	0	0	1	1	1	1	10
MICAL3	1	1	0	1	0	1	1	1	1	0	0	1	1	1	10
PLCH1	1	1	1	1	0	1	1	1	0	0	0	1	1	1	10
F5	1	1	1	1	0	1	1	1	0	0	0	1	1	1	10
LPHN3	1	1	1	1	0	1	1	1	0	0	0	1	1	1	10
ZNF91	1	1	1	1	0	1	1	1	0	0	0	1	1	1	10
USP24	1	1	1	1	0	1	1	1	0	0	0	1	1	1	10
FLNC	1	1	1	1	0	1	1	1	0	0	0	1	1	1	10
PRRC2B	1	1	1	1	0	0	0	1	1	0	1	1	1	1	10
NAV1	1	1	1	1	0	1	1	1	0	0	0	1	1	1	10
KNDC1	1	1	0	1	0	1	1	1	0	0	1	1	1	1	10
BRIP1	1	1	1	1	0	1	0	1	0	0	1	1	1	1	10
WNK4	1	1	1	1	0	1	0	1	0	1	0	1	1	1	10
DENND4A	1	1	1	1	0	1	1	1	0	0	0	1	1	1	10
COL27A1	1	1	0	1	0	1	1	1	1	0	0	1	1	1	10
DNMT3A	1	1	1	1	0	1	1	1	0	0	0	1	1	1	10

MIA3	1	1	1	1	0	1	1	1	0	0	0	1	1	1	10
KDM2B	1	1	1	1	0	1	1	1	0	0	0	1	1	1	10
ARHGEF11	1	1	1	1	0	1	1	1	0	0	0	1	1	1	10
GPR179	1	1	1	1	0	1	1	1	0	0	0	1	1	1	10
NOTCH4	1	1	1	1	0	1	1	1	0	0	0	1	1	1	10
ITGAL	1	1	1	1	0	1	1	1	0	0	0	1	1	1	10
FLNA	1	1	1	1	0	1	1	1	0	0	0	1	1	1	10
ADCY8	1	1	1	1	0	1	1	1	0	0	0	1	1	1	10
NOS2	1	1	1	1	0	1	1	1	0	0	0	1	1	1	10
SETD1A	1	1	0	1	0	1	1	1	0	1	0	1	1	1	10
SLITRK5	1	1	0	1	0	1	0	1	1	1	0	1	1	1	10
ANKRD30A	1	1	0	1	0	1	1	1	0	1	0	1	1	1	10
MCM3AP	1	1	0	1	0	1	1	1	0	1	0	1	1	1	10
PALB2	1	1	1	1	0	1	0	1	0	0	1	1	1	1	10
ARID1A	1	1	1	1	0	1	1	1	0	0	0	1	1	1	10
PCF11	1	1	1	1	0	1	1	1	0	0	0	1	1	1	10
NUP214	1	1	1	1	0	1	1	1	0	0	0	1	1	1	10
DNAH1	1	1	1	1	0	1	0	1	0	0	1	1	1	1	10
ITPR3	1	1	1	1	0	1	1	1	0	0	0	1	1	1	10
ZEB2	1	1	1	1	0	0	1	1	1	0	0	1	1	1	10
PDE10A	1	1	1	1	0	1	1	1	0	0	0	1	1	1	10
PTPRM	1	1	1	1	0	1	1	1	0	0	0	1	1	1	10
KIF19	1	1	1	1	0	1	1	1	0	0	0	1	1	1	10
MYO3A	1	1	0	1	0	1	1	1	0	1	0	1	1	1	10
ITPR1	1	1	1	1	0	1	1	1	0	0	0	1	1	1	10

PLXNC1	1	1	1	1	0	1	1	1	0	0	0	1	1	1	10
EHMT1	1	1	1	1	0	1	1	1	0	0	0	1	1	1	10
PREX2	1	1	1	1	0	1	1	1	0	0	0	1	1	1	10
SYMPK	1	1	1	1	0	1	1	1	0	0	0	1	1	1	10
CUL9	1	1	1	1	0	0	1	1	0	0	1	1	1	1	10
CNOT1	1	1	0	1	0	1	1	1	1	0	0	1	1	1	10
DNAH2	1	1	1	1	0	1	1	1	0	0	0	1	1	1	10
IQGAP1	1	1	0	1	0	1	1	1	1	0	0	1	1	1	10
SMARCC2	1	1	1	1	0	0	1	1	0	1	0	1	1	1	10
JARID2	1	1	1	1	0	1	1	1	0	0	0	1	1	1	10
NKTR	1	1	1	1	0	0	1	1	0	1	1	0	1	1	10
RPS6KC1	1	1	1	1	0	1	1	1	0	0	0	1	1	1	10
AUTS2	1	1	1	1	0	1	0	1	0	0	1	1	1	1	10
DOPEY1	1	1	1	1	0	1	1	1	0	0	0	1	1	1	10
SUPT16H	1	1	1	1	0	1	0	1	1	0	0	1	1	1	10
ARHGAP35	1	1	0	1	0	1	1	1	0	0	1	1	1	1	10
WDR64	1	1	1	1	0	0	1	1	0	0	1	1	1	1	10
NLRP2	1	1	1	1	0	1	1	1	0	0	0	1	1	1	10
MADD	1	1	1	1	0	1	1	1	0	0	0	1	1	1	10
TRPM3	1	1	0	1	0	1	0	1	1	0	1	1	1	1	10
POLR1A	1	1	1	1	0	0	1	1	1	0	0	1	1	1	10
GREB1	1	1	1	1	0	1	1	1	0	0	0	1	1	1	10
FAM135B	1	1	1	1	0	1	0	1	1	0	0	1	1	1	10
MYO9A	1	1	1	1	0	1	1	1	0	0	0	1	1	1	10
NSD1	1	1	1	1	0	1	1	1	0	0	0	1	1	1	10

SCN4A	1	1	1	1	0	1	1	1	0	0	0	1	1	1	10
CUL7	1	1	1	1	0	1	1	1	0	0	0	1	1	1	10
INTS1	1	1	1	1	0	1	1	1	0	0	0	1	1	1	10
RBBP6	1	1	0	1	0	1	1	1	0	0	1	1	1	1	10
MYO10	1	1	1	1	0	1	1	1	0	0	0	1	1	1	10
NBEA	1	1	1	1	0	1	1	1	0	0	0	1	1	1	10
IFT172	1	1	1	1	0	1	1	1	0	0	0	1	1	1	10
PLCZ1	1	1	0	1	0	1	1	1	0	0	1	1	1	1	10
PKD1L1	1	1	0	1	0	1	1	1	0	0	1	1	1	1	10
FANCM	1	1	0	1	0	1	1	1	0	1	0	1	1	1	10
CDH23	1	1	1	1	0	1	1	1	0	0	0	1	1	1	10
THADA	1	1	1	1	0	1	0	1	1	0	0	1	1	1	10
PLEKHG4B	1	1	1	1	0	1	1	1	0	0	0	1	1	1	10
MYH7	1	1	1	1	0	1	1	1	0	0	0	1	1	1	10
GABRG3	1	1	1	1	0	1	1	1	0	0	0	1	1	1	10
KRT75	1	1	1	1	0	1	1	1	0	0	0	1	1	1	10
AKAP6	1	1	1	1	0	1	1	1	0	0	0	1	1	1	10
DOCK6	1	1	1	1	0	1	0	1	0	0	1	1	1	1	10
ADAMTS5	1	1	1	1	0	1	1	1	0	0	0	1	1	1	10
NAALAD2	1	1	0	1	0	1	1	1	0	0	1	1	1	1	10
KIF13B	1	1	1	1	0	1	1	1	0	0	0	1	1	1	10
CACNA1G	1	1	1	1	0	1	1	1	0	0	0	1	1	1	10
EPHA5	1	1	1	1	0	1	0	1	1	0	0	1	1	1	10
RALGAPB	1	1	1	1	0	1	1	1	0	0	0	1	1	1	10
LAMA4	1	1	1	1	0	1	0	1	1	0	0	1	1	1	10

DOCK11	1	1	1	1	0	1	1	1	0	0	0	1	1	1	10
SMARCA4	1	1	0	1	0	1	1	1	0	0	1	1	1	1	10
TMTC3	1	1	1	1	0	1	1	1	0	0	0	1	1	1	10
PAPPA2	1	1	1	1	0	1	0	1	1	0	0	1	1	1	10
BANK1	1	1	1	1	0	1	1	1	0	0	0	1	1	1	10
TTC40	1	1	1	1	0	1	1	1	0	0	0	1	1	1	10
DENND2A	1	1	1	1	0	0	1	1	0	0	1	1	1	1	10
CADPS2	1	1	0	1	0	1	1	1	0	0	1	1	1	1	10
FAT1	1	1	1	1	0	1	1	1	0	0	0	1	1	1	10
EPB41L3	1	1	1	1	0	1	0	1	0	1	0	1	1	1	10
NOTCH2	1	1	1	1	0	0	1	1	0	1	0	1	1	1	10
SLIT3	1	1	1	1	0	1	0	1	0	0	1	1	1	1	10
ZNF208	1	1	0	1	0	1	1	1	1	0	0	1	1	1	10
TDRD6	1	1	0	1	0	1	1	1	1	0	0	1	1	1	10
KIF16B	1	1	0	1	0	1	1	1	0	0	1	1	1	1	10
CHRM2	1	1	1	1	0	1	1	1	0	0	0	1	1	1	10
ZNF804A	1	1	0	1	0	1	1	1	1	0	0	1	1	1	10
ACIN1	1	1	1	1	0	1	1	1	0	0	0	1	1	1	10
NAA16	1	1	1	1	0	1	1	1	0	0	0	1	1	1	10
PCM1	1	1	0	1	0	1	1	1	0	0	1	1	1	1	10
LEPR	1	1	1	1	0	1	1	1	0	0	0	1	1	1	10
ZDBF2	1	1	1	1	0	1	1	1	0	0	0	1	1	1	10
SPEG	1	1	1	1	0	1	1	1	0	0	0	1	1	1	10
RANBP6	1	1	1	1	0	1	0	1	0	1	0	1	1	1	10
NFATC3	1	1	1	1	0	1	1	1	0	0	0	1	1	1	10

ASCC3	1	1	1	1	0	1	1	1	0	0	0	1	1	1	10
CNTNAP5	1	1	1	1	0	1	1	1	0	0	0	1	1	1	10
NIN	1	1	1	1	0	1	0	1	1	0	0	1	1	1	10
TRERF1	1	1	1	1	0	1	1	1	0	0	0	1	1	1	10
TRPC6	1	1	1	1	0	1	1	1	0	0	0	1	1	1	10
PTPRT	1	1	1	1	0	1	0	1	1	0	0	1	1	1	10
NOD2	1	1	0	1	0	1	1	1	1	0	0	1	1	1	10
COL6A3	1	1	1	1	0	1	1	1	0	0	0	1	1	1	10
CSMD1	1	1	1	1	0	1	1	1	0	0	0	1	1	1	10
AKAP4	1	1	1	1	0	1	1	1	0	0	0	1	1	1	10
ZMYM4	1	1	0	1	0	0	1	1	0	1	1	1	1	1	10
DCC	1	1	1	1	0	1	0	1	0	0	1	1	1	1	10
BRWD3	1	1	1	1	0	1	1	1	0	0	0	1	1	1	10
SEC31A	1	1	1	1	0	0	1	1	0	1	0	1	1	1	10
ANO4	1	1	1	1	0	1	0	1	1	0	0	1	1	1	10
CELSR1	1	1	1	1	0	1	1	1	0	0	0	1	1	1	10
TSHZ3	1	1	0	1	0	1	1	1	1	0	0	1	1	1	10
EYS	0	1	1	1	1	1	0	1	0	0	1	1	1	1	10
DUOX1	0	1	1	1	0	1	1	1	0	0	1	1	1	1	10
CHD3	0	1	1	1	0	1	1	1	0	1	0	1	1	1	10
KIAA1432	0	1	1	1	0	1	0	1	1	1	0	1	1	1	10
CELSR2	0	1	1	1	0	1	1	1	1	0	0	1	1	1	10
MAP3K4	0	1	1	1	0	1	1	1	0	1	0	1	1	1	10
PXDNL	0	1	1	1	0	1	1	1	1	0	0	1	1	1	10
KIAA1244	0	1	1	1	0	1	1	1	0	0	1	1	1	1	10

NBAS	0	1	1	1	0	1	1	1	1	0	0	1	1	1	10
AFF3	0	1	1	1	0	1	1	1	0	1	0	1	1	1	10
INF2	0	1	1	1	0	0	1	1	0	1	1	1	1	1	10
FLT1	0	1	1	1	0	1	1	1	1	0	0	1	1	1	10
BZRAP1	0	1	1	1	0	1	1	1	0	1	0	1	1	1	10

Note: "**1**" indicates that TMB significantly higher in gene-mutated type than in gene-wildtype tumors (two-sided Student's t test, FDR<0.1);

"**0**" indicates that TMB not significantly higher in gene-mutated type than in gene-wildtype tumors;

"**Total**" indicates the number of cancer types in which the TMB significantly higher in gene-mutated type than in gene-wildtype tumors.

Table S1B. 28 genes whose mutations correlated with reduced TMB in a single cancer type.

Gene symbol	BLCA	BRCA	COAD	GBM	LUAD	SKCM	Total
LA16c-23H5.4	1	0	0	0	0	0	1
FGFR3	1	0	0	0	0	0	1
MAP3K1	0	1	0	0	0	0	1
GATA3	0	1	0	0	0	0	1
APC	0	0	1	0	0	0	1
IDH1	0	0	0	1	0	0	1
EGFR	0	0	0	0	1	0	1
RP11-526A4.1	0	0	0	0	1	0	1
NPIPB11	0	0	0	0	1	0	1
SLC50A1	0	0	0	0	1	0	1
RP11-98J9.2	0	0	0	0	1	0	1
RP11-871F6.3	0	0	0	0	1	0	1
AC026188.1	0	0	0	0	1	0	1
RP11-149A7.2	0	0	0	0	1	0	1
RP11-708B6.2	0	0	0	0	1	0	1
AC079135.1	0	0	0	0	1	0	1
RP11-588H23.3	0	0	0	0	1	0	1
EIF4E	0	0	0	0	1	0	1
AC007246.3	0	0	0	0	1	0	1
RP11-377D9.3	0	0	0	0	1	0	1
DIO2-AS1	0	0	0	0	1	0	1
ACADSB	0	0	0	0	1	0	1
RPS11P6	0	0	0	0	1	0	1
RP11-310I9.1	0	0	0	0	1	0	1
PBX2P1	0	0	0	0	0	1	1
CTD-2281E23.3	0	0	0	0	0	1	1
CTD-3037G24.3	0	0	0	0	0	1	1
EEF1B2	0	0	0	0	0	1	1

Note: "**1**" indicates that TMB significantly lower in gene-mutated type than in gene-wildtype tumors (two-sided Student's t test, FDR<0.1);

"**0**" indicates that TMB not significantly lower in gene-mutated type than in gene-wildtype tumors;

"**Total**" indicates the number of cancer types in which the TMB significantly lower in gene-mutated type than in gene-wildtype tumors.

Table S2. A summary of datasets used in this study.

Dataset	Number of all samples	Source
TCGA-ACC	208	https://portal.gdc.cancer.gov/
TCGA-BLCA	427	
TCGA-BRCA	1212	
TCGA-CESC	316	
TCGA-CHOL	45	
TCGA-COAD	328	
TCGA-ESCA	196	
TCGA-GBM	171	
TCGA-HNSC	566	
TCGA-KICH	91	
TCGA-KIRC	606	
TCGA-KIRP	323	
TCGA-LAML	511	
TCGA-LIHC	423	
TCGA-LUAD	576	
TCGA-LUSC	552	
TCGA-OV	396	
TCGA-PAAD	347	
TCGA-PRAD	550	
TCGA-READ	105	
TCGA-SKCM	1029	
TCGA-STAD	450	
TCGA-TGCT	322	
TCGA-THCA	568	
TCGA-UCEC	381	
TCGA-Pan-cancer	9685	
MSKCC-Samstein cohort	1610	[1]
MSKCC-Zehir cohort	7091	[1]
Rizvi cohort	33	[2]
Hellmann cohort	240	[3]
Allen cohort	110	[4]
Hugo cohort	37	[5]

Table S3. The sample size for high-TMB and low-TMB cancers.

Cancer type	Numbers of high-TMB	Numbers of low-TMB	Rate of high-TMB
Pan-cancer	1055	8127	11%
SKCM	233	239	49%
LUAD	207	354	37%
BLCA	103	293	26%

STAD	75	304	20%
HNSC	66	446	13%
UCEC	58	190	23%
LUSC	50	128	28%
COAD	47	172	21%
BRCA	25	968	3%
ESCA	23	162	12%
CESC	20	179	10%
LIHC	20	353	5%
PAAD	20	165	11%
SARC	12	247	5%
DLBC	9	39	19%
ACC	7	83	8%
GBM	7	309	2%
TGCT	5	151	3%
PRAD	4	495	1%
READ	4	77	5%
LGG	3	280	1%
UCS	2	55	4%
CHOL	1	35	3%
KICH	1	65	2%
THYM	1	122	1%
UVM	1	79	1%
KIRC	0	491	0%
KIRP	0	282	0%
LAML	0	197	0%
OV	0	230	0%
PCPG	0	184	0%
THCA	0	504	0%

Supplementary Table 4. The marker genes of immune signatures and proliferation

Signature	Gene sets
Proliferation	*CCNB1, CDC20, CDKN3, CDK1, MAD2L1, PRC1, RRM2*
Immune cytolytic activity	*PRF1, GZMA*
CD4+ regulatory T cells	*CTLA4, FOXP3, GPR15, IL32, IL4, IL5*
CD8+ T cells	*CD8A*
Pro-inflammatory cytokines	*IFNG, IL1A, IL1B, IL2*
Anti-inflammatory cytokines	*TGFB1, IL10, IL4, IL11*
M1 macrophage	*FCGR1A, IDO1, SOCS1, CXCL10*
M2 macrophage	*MRC1, TGM2, FCER2, CCL22*

References

1. Samstein RM, Lee CH, Shoushtari AN, Hellmann MD, Shen R, Janjigian YY, et al. Tumor mutational load predicts survival after immunotherapy across multiple cancer types. Nat Genet. 2019;51(2):202-6.
2. Rizvi H, Sanchez-Vega F, La K, Chatila W, Jonsson P, Halpenny D, et al. Molecular Determinants of Response to Anti-Programmed Cell Death (PD)-1 and Anti-Programmed Death-Ligand 1 (PD-L1) Blockade in Patients With Non-Small-Cell Lung Cancer Profiled With Targeted Next-Generation Sequencing. J Clin Oncol. 2018;36(7):633-41.
3. Rizvi NA, Hellmann MD, Snyder A, Kvistborg P, Makarov V, Havel JJ, et al. Cancer immunology. Mutational landscape determines sensitivity to PD-1 blockade in non-small cell lung cancer. Science (New York, NY). 2015;348(6230):124-8.
4. Van Allen EM, Miao D, Schilling B, Shukla SA, Blank C, Zimmer L, et al. Genomic correlates of response to CTLA-4 blockade in metastatic melanoma. Science (New York, NY). 2015;350(6257):207-11.
5. Hugo W, Zaretsky JM, Sun L, Song C, Moreno BH, Hu-Lieskovan S, et al. Genomic and Transcriptomic Features of Response to Anti-PD-1 Therapy in Metastatic Melanoma. Cell. 2016;165(1):35-44.

Supplementary Figures

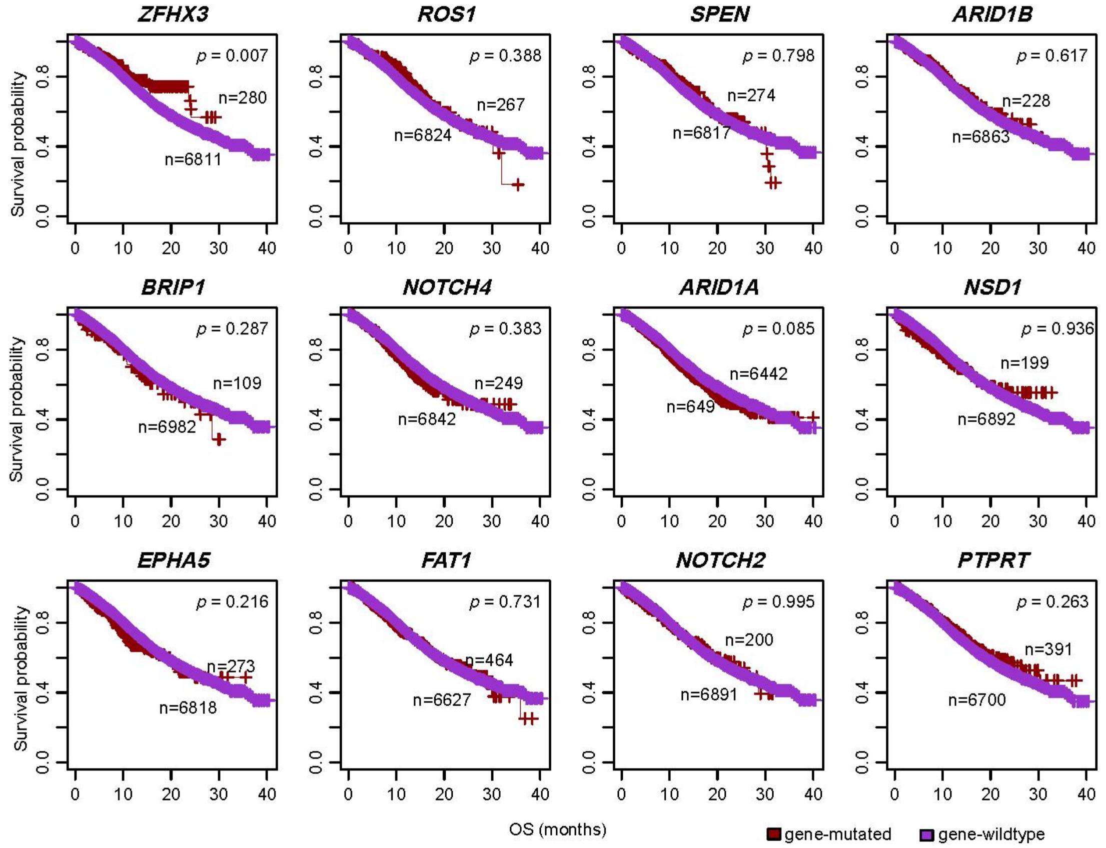

Figure S1. Kaplan-Meier curves show that the mutations in certain genes (expect *ZFHX3*) whose mutations were significantly associated with increased TMB have no significant correlation with overall survival in pan-cancer (MSKCC-Zehir cohort) not receiving the ICB therapy.

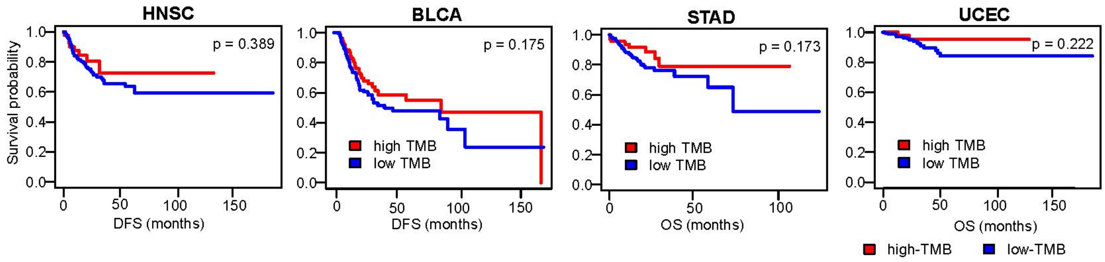

Figure S2. The association between TMB and survival prognosis in different cancer types.

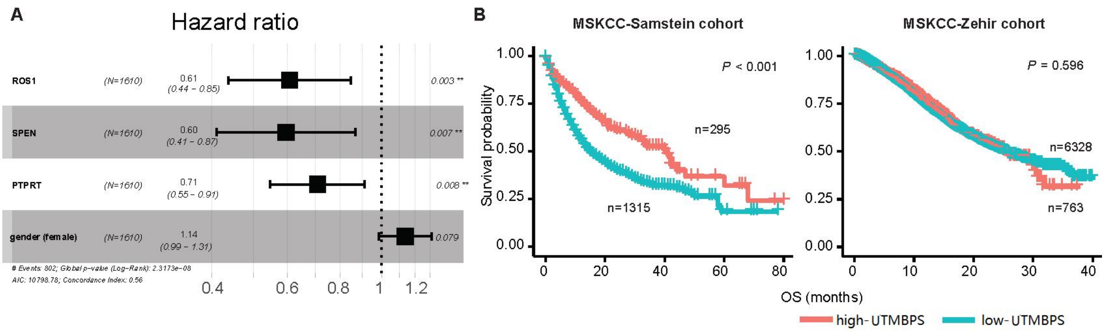

Figure S3. Correlations between mutations of three genes, gender and OS. A. Cox proportional hazards model in the MSKCC-Samstein cohort. **B**. Kaplan-Meier curves showing that the upgraded TMB prognostic score (UTMBPS) correlates positively with OS in the MSKCC-Samstein cohort receiving the ICB therapy, while it shows no significant correlation with OS in the MSKCC-Zehir cohort without the ICB therapy.
